# Reversible Thermal Conductivity Switching Using Flexible Metal–Organic Frameworks

**DOI:** 10.1021/acs.chemmater.3c00496

**Published:** 2023-08-02

**Authors:** Hasan Babaei, Katie R. Meihaus, Jeffrey R. Long

**Affiliations:** † Department of Chemistry, University of California, Berkeley, Berkeley, California 94720, United States; ‡ Department of Chemical and Biomolecular Engineering, University of California, Berkeley, Berkeley, California 94720, United States; § Materials Sciences Division, Lawrence Berkeley National Laboratory, Berkeley, California 94720, United States

## Abstract

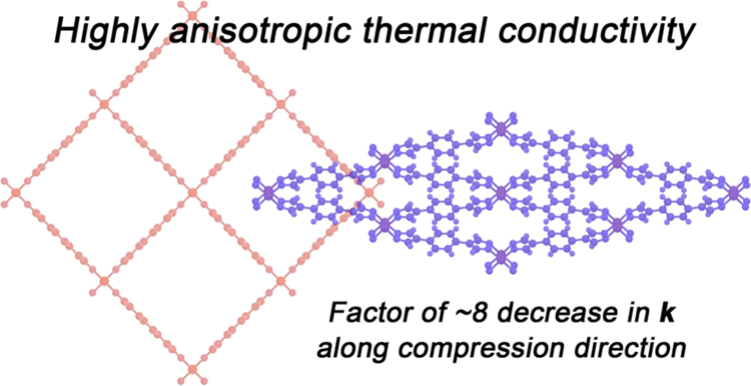

The ability to control thermal transport is critical for the design of thermal rectifiers, logic gates, and transistors, although it remains a challenge to design materials that exhibit large changes in thermal conductivity with switching ratios suitable for practical applications. Here, we propose the use of flexible metal–organic frameworks, which can undergo significant structural changes in response to various stimuli, to achieve tunable switchable thermal conductivity. In particular, we use molecular dynamics simulations to show that the thermal conductivity of the flexible framework Fe(bdp) (bdp^2–^ = 1,4-benzenedipyrazolate) becomes highly anisotropic upon transitioning from the expanded to the collapsed phase, with the conductivity decreasing by nearly an order of magnitude along the direction of compression. Our results add to a small but growing number of studies investigating metal–organic frameworks for thermal transport.

## Introduction

Switchable thermal transport is key to the development of rectifiers, transistors, switches, and rheostats that operate based on heat transfer.^1^ Realization of these devices would enable a revolution in the thermal management of electronics, solid-state cooling, and energy conversion,^2−4^ but such advances hinge on the development of materials wherein thermal conductivity can be controlled in a predictable way. A thermal conductivity switch is characterized by several parameters, including the degree to which the conductivity changes upon switching (switching ratio), how fast switching occurs (switching time), the external stimulus used to trigger the change, and cyclability.

A number of materials have been shown to exhibit switchable thermal transport, with switching ratios typically ranging from 1.5 to 5 on time scales that range from seconds to minutes.^1,5−12^ For example, light-induced switching between *trans* and *cis* conformations of an azobenzene-based polymer results in a reversible change in thermal conductivity by a factor of 3.^13^ The switching, which is also associated with a crystal-to-liquid transformation, occurs within tens of seconds at room temperature. It has also been shown that hydration of topologically networked bio-polymers can lead to reversible switching of thermal conductivity by a factor of 4, with reported cycles indicating a switching time of a few minutes.^5^ In another study, it was shown that the modulation of liquid crystal alignment with a magnetic field above the material glass-transition temperature can give rise to a change in thermal conductivity by a factor of 1.5 over the course of 10 min.^12^ Interestingly, in the case of radiative heat transfer, it has also been shown that placing a planar object in the vicinity of two coplanar SiN membranes can give rise to changes in thermal transport by a factor as large as 5, on a time scale of about 2 min.^7^ Despite progress in this area, it remains a challenge to design materials that exhibit suitably large (order of magnitude) switching ratios on time scales that are fast enough for practical devices. Notably, it was recently shown that a thermally induced structural phase transition in crystalline polyethylene nanofibers results in a large, reversible change in thermal conductance associated with a switching ratio of ∼10 that is the record for any solid–solid or solid–liquid phase change.^14^

It is clear that the pursuit of materials exhibiting a strong, well-defined structural change in response to external stimuli is a promising route to achieving facile thermal switchability over large conductivity scales. In this context, metal–organic frameworks (MOFs) stand as intriguing candidate materials. Constructed from metal nodes and multitopic organic linkers,^15^ MOFs are porous, crystalline solids that are of interest for a variety of applications, including gas storage, molecular separations, and catalysis,^15−19^ and their ostensibly simple construction affords access to an almost unlimited number of structures with diverse properties. In particular, in response to external stimuli such as temperature,^20^ pressure,^21^ or host–guest interactions,^22,23^ certain frameworks exhibit structural flexibility and reversible phase transformations that result in substantial contraction or expansion of their pores. Mechanisms of phase transitions in flexible MOFs have been studied in detail,^24−30^ and step-shaped adsorption profiles associated with guest-induced transformations have rendered these materials promising for various storage^19^ and separation applications.^31^ For example, the flexible framework Co(bdp) (bdp^2–^ = 1,4-benzenedipyrazolate)^32^ is capable of storing large quantities of methane at moderate temperatures and pressures in response to a reversible phase transition that also affords the material with intrinsic heat management,^22^ and this transition can be tuned based on the choice of metal or linker substituent.^33^ A very different phase change in this same material in the presence of carbon dioxide gives rise to near-perfect selectivity for CO_2_ over CH_4_.^31^ Previous experimental^34−41^ and computational^38,42−55^ investigations of thermal transport in MOFs have predominantly been limited to rigid MOFs, and only two recent computational studies have investigated heat transfer in flexible MOFs with an emphasis on adsorption-based applications.^56,57^

**Figure 1:**
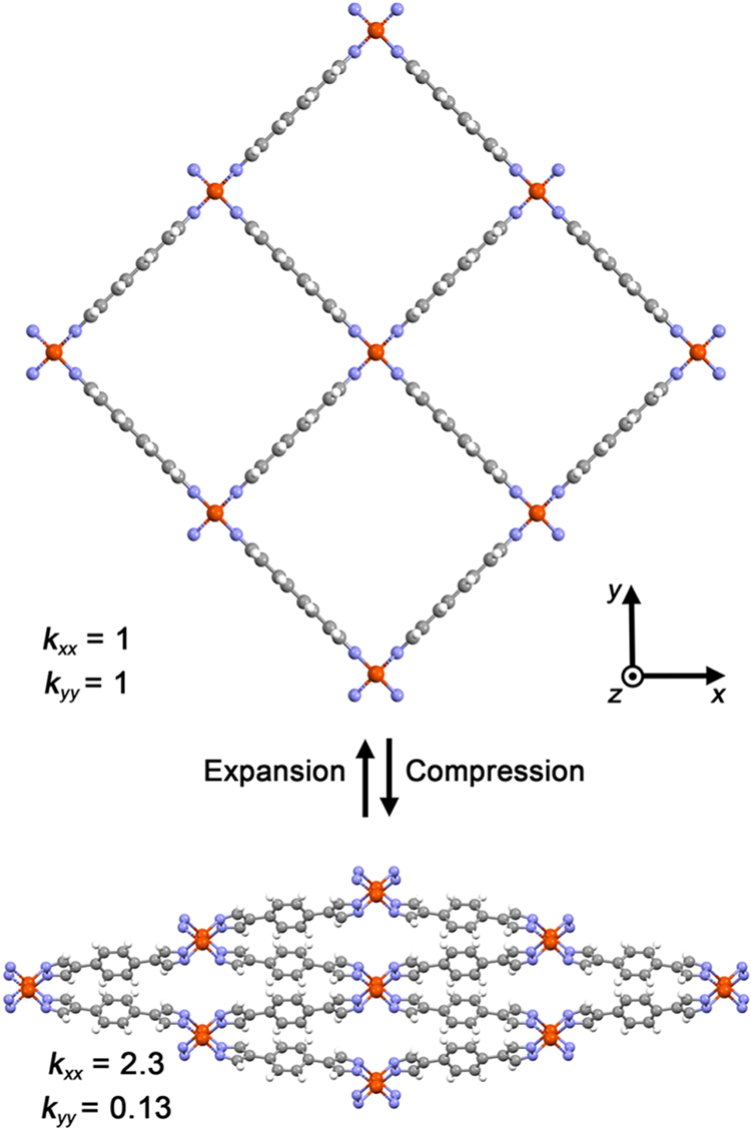
(Top) Powder X-ray diffraction structure of expanded Fe(bdp)^22^ viewed down the framework channel and diagonal elements of thermal conductivity tensor in the *x* and *y* directions. (Bottom) Powder X-ray diffraction structure of compressed Fe(bdp)^22^ and associated thermal conductivity values along the *x* and *y* directions. For both structures, *k*_*zz*_ = 0.17. The unnormalized value of thermal conductivity for the expanded framework in the *xx* direction is 4.6 W/mK. All thermal conductivity values are normalized to the thermal conductivity of expanded Fe(bdp) in the *x* direction. Red, blue, gray, and white spheres represent Fe, N, C, and H atoms, respectively. Thermal transport in Fe(bdp) was investigated in this work using molecular dynamics simulations.

Here, we use molecular dynamics (MD) simulations to study thermal transport in the flexible material Fe(bdp),^22^ and we apply the Green–Kubo method to predict thermal conductivity before and after framework contraction. Consistent with previous studies,^56,57^ we find the thermal conductivity of the material changes upon transitioning from the expanded to the collapsed phase. In particular, the thermal conductivity decreases by nearly an order of magnitude (∼8) along *y*, the direction of framework compression, whereas it increases by a factor of ∼2 along the *x* direction (Figure 1). We also calculate the thermal conductivity tensor as a function of crystallographic direction and find that the highest thermal conductivity change overall occurs in the *y* direction, which is one of the principal directions of the thermal conductivity tensor.

## Methods

We calculated the thermal conductivity of Fe(bdp) before and after framework contraction. The simulation cell size for both expanded and contracted frameworks was set based on the experimental lattice constants, available from the structures reported in ref (22). For the interactions between atoms in Fe(bdp), we used a modified version of the DREIDING force field,^58^ in which the equilibrium distances between atoms were adjusted to the experimental structures. This force field has previously been used to simulate other properties of MOFs, such as bulk modulus and thermal expansion.^59^ The Green–Kubo method was used to predict thermal conductivity.^60^ This method involves calculating the instantaneous heat flux in an equilibrium MD simulation.

The MD simulations were performed using a version of the Large-scale Atomic/Molecular Massively Parallel Simulator^61^ software, which can correctly implement heat flux for many-body potentials.^62^ Details of the Green–Kubo calculations for thermal conductivity are provided in the Supporting Information (SI). For determining thermal conductivity, we used a system size of 4 × 4 × 4 unit cells (in crystallographic directions *a*, *b*, and *c*, corresponding to *x*, *y*, and *z* directions in Figure 1). See Figure 1 for snapshots of the simulation cells for Fe(bdp) before and after contraction. In the case of expanded Fe(bdp), the system was initially equilibrated under *NPT* (constant pressure–constant temperature) conditions at a temperature of 300 K and atmospheric pressure for 500,000 time steps; under *NVT* (constant volume–constant temperature) conditions at a temperature of 300 K for 300,000 time steps; and for 200,000 time steps under *NVE* (constant volume–constant energy) conditions. Finally, *NVE* simulations were run for an additional 5,000,000 time steps where the heat current was calculated every five time steps.

## Results and Discussion

The predicted thermal conductivities along the *x*, *y*, and *z* directions, i.e., the diagonal elements of the thermal conductivity tensor ***k*** (*k*_*xx*_, *k*_*yy*_, and *k*_*zz*_), normalized by the thermal conductivity in the *x*-direction of expanded Fe(bdp), are given in Figure 1. In expanded Fe(bdp), the thermal conductivities in the *x* and *y* directions are the same, revealing isotropy in the *xy*-plane. However, the thermal conductivity in the *z* direction is nearly an order of magnitude smaller than in the *x* and *y* directions. The thermal conductivity in the *z* direction does not change upon contraction of Fe(bdp), but *k*_*yy*_ decreases substantially by a factor of 7.7, and *k*_*xx*_ increases by a factor of 2.3. The significant change in thermal conductivity accompanying the material phase change indicates that, in addition to other applications of flexible MOFs, these materials could be used as thermal conductivity switches.

The changes in thermal conductivity can be understood by considering that compression decreases the alignment of the framework bonds along the *y* direction, which makes them less effective in transferring heat, whereas it increases the bond alignment along the *x* direction, leading to more effective heat transfer. Note that there are a number of additional factors that likely play a role in modulating relative thermal conductivities. For example, as demonstrated previously for Co(bdp),^22,33^ in the collapsed phase of M(bdp), there are edge-to-face π–π interactions between the benzene rings of adjacent linkers, which may result in stronger phonon scatterings and lower thermal conductivity; on the other hand, these interactions could provide additional pathways for heat transfer along the *x* direction. It is also relevant to note that the phonon scattering rates are dependent on the symmetry of the crystal, and in the case of Fe(bdp), the expanded phase has a higher symmetry than the collapsed phase. Other factors such as an increase in the stiffness of the linker (for example, if compression does not occur under constant pressure) and framework density in the collapsed phase may result in improved heat transfer. Examining the effects of these changes would require evaluating phonon scattering rates, which is computationally infeasible for a full MOF structure, given the large number of atoms in the unit cell. However, previous studies on idealized structures, wherein atoms were grouped in a representative smaller number of interaction sites, suggest that effects related to phonon scattering do not lead to significant changes in thermal conductivity.^47,48^

The off-diagonal elements of ***k*** (*k*_*ij*_, *i,j* = *x*, *y*, and *z*) were also calculated for expanded and collapsed Fe(bdp) using the Green–Kubo method, all of which were found to be zero. Thus, the *x*, *y*, and *z* directions are the principal directions of ***k***. This information is critical for practical materials development, for instance, in identifying materials for which crystals can be grown and aligned in the direction of the greatest thermal conductivity change.

**Figure 2:**
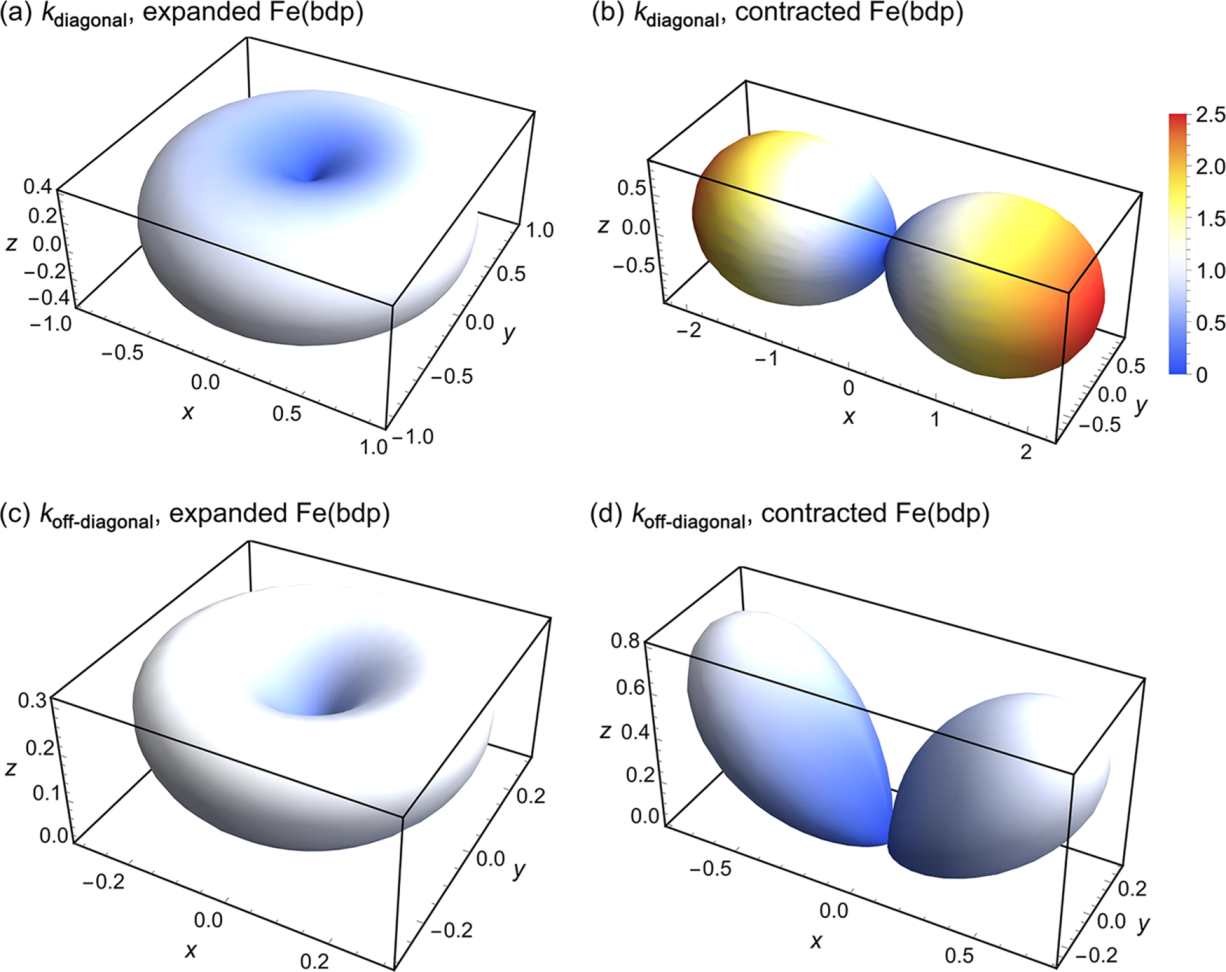
Calculated three-dimensional contours of directionally dependent diagonal and off-diagonal elements of thermal conductivity tensor for expanded (a, c) and contracted (b, d) Fe(bdp) in the *xyz* coordinate system. The off-diagonal element plotted in (c) and (d) is *k*_*xz*_. These objects can be interpreted as follows: the distance from the center of the coordinate system (*x* = *y* = *z* = 0) to any point on the surface of the object is the thermal conductivity in that direction. The radial distances and colors represent thermal conductivity magnitudes. Thermal conductivities are normalized by the diagonal element of the thermal conductivity tensor of expanded Fe(bdp) along the *x* direction, *k*_*xx*_. See Figure S2 for the three-dimensional contours of the tensor elements *k*_*xy*_ and *k*_*yz*_ transformed by the transformation matrix, for the contracted framework. For expanded Fe(bdp), transformed *k*_*xy*_ is zero and transformed *k*_*yz*_ is the same as *k*_*xz*_ due to isotropy in the *xy* plane.

We next sought to examine the thermal conductivity in all other crystallographic directions for expanded and collapsed Fe(bdp). Accordingly, the thermal conductivity for both phases was calculated as a function of polar and azimuthal angles in the spherical coordinate system. We transformed the thermal conductivity tensor ***k***^*xyz*^ in the *xyz* coordinate system to the thermal conductivity tensor ***k***^*x′y′z′*^ in the *x′y′z′* coordinate system, where *x′*, *y′*, and *z′* axes are obtained by rotating the *x*, *y*, and *z* axes through polar angle (θ) and azimuthal angle (φ) rotations. Using Fourier’s law ***q***^*xyz*^ = −***k***^*xyz*^∇*T* and ***q***^*x′y′z′*^ = −***k***^*x′y′z′*^∇′*T* (where ***q***^*xyz*^ and ***q***^*x′y′z′*^ are the heat flux vectors in the *xyz* and *x′y′z′* coordinate systems, respectively) and the rotation matrices **R**(θ) and **R**(φ), the coordinate transformation leads to ***k***^*x′y′z′*^ = **A*****k***^*xyz*^**A**^T^, where **A** = **R**(θ)**R**(φ) and **A**^T^ is the transpose of **A**. Figure 2 shows the three-dimensional contours of select directionally dependent elements of the thermal conductivity tensor ***k*** for the expanded (Figure 2a,c, diagonal and off-diagonal elements, respectively) and contracted (Figure 2b,d, diagonal and off-diagonal elements, respectively) frameworks.

The off-diagonal element shown in Figure 2c,d corresponds to *k*_*xz*_ transformed by the transformation matrix. Due to isotropy in the *xy* plane (*k*_*xx*_ = *k*_*yy*_ ≠ *k*_*zz*_), the three-dimensional thermal conductivity for the expanded framework is donut-shaped, while for the contracted framework, the three-dimensional thermal conductivity is dumbbell-shaped, due to the anisotropy in the original *xyz* directions (*k*_*xx*_ ≠ *k*_*yy*_ ≠ *k*_*zz*_). The highest and lowest diagonal-element thermal conductivities for both frameworks occur in the original *x*, *y*, and *z* directions (see Figure 1), which construct the principal direction of ***k***. In particular, for the contracted framework, the highest and lowest diagonal elements occur in the *x* and *y* directions. In contrast, for the expanded framework, the highest thermal conductivity occurs in the *x* and *y* directions and the lowest thermal conductivity occurs in the *z* direction. Moreover, since the thermal conductivities in principal directions *x* and *y* are the same, thermal conductivity is constant in all directions of the *xy*-plane. Overall, the three-dimensional off-diagonal term for the contracted framework is larger than that of the expanded framework, due to the higher anisotropy of the thermal conductivity for the contracted form in the principal directions (*xyz*). We note that the transformation of the off-diagonal terms involves two directions and is therefore more complex than the transformation of the diagonal terms. One direction corresponds to the heat flow and the other corresponds to the temperature gradient. Plots for the *k*_*xz*_ off-diagonal term are illustrated in Figure 2c,d; the plots for the transformation of the other off-diagonal terms are shown in the SI.

In addition to the high switching ratio of Fe(bdp), the highly anisotropic thermal conductivity of the contracted phase and the resulting high value of the off-diagonal elements (see Figure S2) could be of great interest for active thermal management. In particular, the nonzero off-diagonal elements would make it possible to redirect heat when needed, or bend heat flux, e.g., a temperature gradient along *x* could be used to create heat flux along *z* where no temperature gradient is present.

It should be noted that, experimentally, the expanded Fe(bdp) structure is only accessible in the presence of guest molecules. In this case, the scattering of framework phonons resulting from collisions between the molecules and the framework pores will give rise to a smaller thermal conductivity in all directions than that calculated for the hypothetical guest-free expanded phase.^38,46,56^ Consequently, the actual switching ratio for Fe(bdp) along *y* will be smaller than predicted, while the switching ratio along *x* will be larger than predicted, with the values depending on the identity of the adsorbed guest. In principle, by changing the nature of the adsorbed guest, it would be possible to modulate the switching ratio achievable with Fe(bdp).

Another intriguing scenario is one in which a flexible framework is capable of undergoing a phase transition in the presence of guests as well as other external stimuli. In this case, it may be possible to tune the thermal conductivity to an even greater degree than is possible for a solely guest-responsive framework.^28^ Consider the flexible framework Al(OH)(bdc) (MIL-53(Al); bdc^2–^ = 1,4-benzenedicarboxylate), which undergoes a guest-induced contraction of 35% upon uptake of water molecules as well as a contraction upon cooling under vacuum.^24^ We carried out MD simulations on MIL-53(Al), which indicate a similar switching behavior for the guest-free framework as found for Fe(bdp). Specifically, the thermal conductivity of guest-free MIL-53(Al) decreases by a factor of ∼5 along the direction of compression, upon going from the expanded framework structure to a collapsed phase (with the same structure as that formed upon adsorption of water), consistent with trends from prior theoretical studies on MIL-53(Al) and other flexible porous materials.^52,53^ Considering the effect of adsorbates on thermal transport in MOFs as discussed above,^38,46,56^ the presence of water in the pores of collapsed MIL-53(Al) may contribute to a further reduction in the thermal conductivity along the direction of compression. Altogether, our results suggest that by combining selective pore contraction and guest–framework interactions in MOFs, it may be possible to modulate thermal conductivity by more than an order of magnitude. Further computational and experimental efforts will be needed to test this hypothesis, including calculations of thermal conductivities in the presence of guests for known flexible frameworks, as well as the design of new flexible MOFs exhibiting structural changes in response to numerous stimuli.

Finally, two additional comments are warranted. First, the foregoing calculations represent results for a perfect crystalline structure. In practice, to achieve large thermal conductivity changes in the bulk material, it will be necessary to synthesize single crystals grown along the direction associated with the largest change or to synthesize thin films or discrete nanocrystals grown with specific crystal faces that can be put in direct contact with a device. MOFs in the form of pellets or powders may not be useful for specific applications. Second, as discussed briefly above, there are multiple possible means of triggering expansion and contraction in a flexible MOF, including the use of guest molecules, temperature, or pressure changes. For instance, contraction can be induced through the application of mechanical pressure via piezoelectric stimulation.^63^ The time scale for switching is critical in applications and depends on the type of trigger; e.g., large density changes achieved through the application of piezoelectric stimulation can give rise to switching frequencies above 10 Hz.^63^ Moreover, MOFs containing photoswitchable bridging ligands, such as azobenzene derivatives, could be adopted to enable the use of light to provide a contactless stimulus with even quicker switching.

## Conclusions

In summary, we have used MD simulations to investigate switchable thermal transport in the flexible framework Fe(bdp). We find that upon contraction, the thermal conductivity decreases by nearly an order of magnitude in the *y* direction (∼8), whereas it more than double in the *x* direction. Moreover, we find that for the contracted framework, the off-diagonal elements of the thermal conductivity tensor can be significant. These results point to other possible applications of these flexible frameworks as thermal conductivity switches in thermal rectifiers, logic gates, or transistors and for active thermal management for energy storage and conversion. Future efforts will be aimed at the large-scale screening of existing and hypothetical flexible MOF structures to identify structural changes and crystal orientations that give rise to the largest switching ratios.

## Supporting Information Available

Additional information on structures, simulation methodology, and thermal conductivity values (PDF)
