# Supplementary material for: Reversible Thermal Conductivity Switching Using Flexible Metal–Organic Frameworks
Source: Chem Mater. 2023 Aug 2;35(16):6220–6. doi: 10.1021/acs.chemmater.3c00496 (PMC10449012; doi:10.1021/acs.chemmater.3c00496)
Supplement: Supplementary file 1 — cm3c00496_si_001.pdf [file cm3c00496_si_001.pdf]

Supporting Information

Reversible Thermal Conductivity Switching Using Flexible Metal-Organic Frameworks

Hasan Babaei,[a]* Katie R. Meihaus,[a] and Jeffrey R. Long[a,b,c]*

[a]Department of Chemistry, University of California, Berkeley, California 94720, United States

[b]Department of Chemical and Biomolecular Engineering, University of California, Berkeley, California 94720, United States

[c]Materials Sciences Division, Lawrence Berkeley National Laboratory, Berkeley, California 94720, United States

*Jeffrey R. Long: jrlong@berkeley.edu; Hasan Babaei: hasan.babaei@berkeley.edu

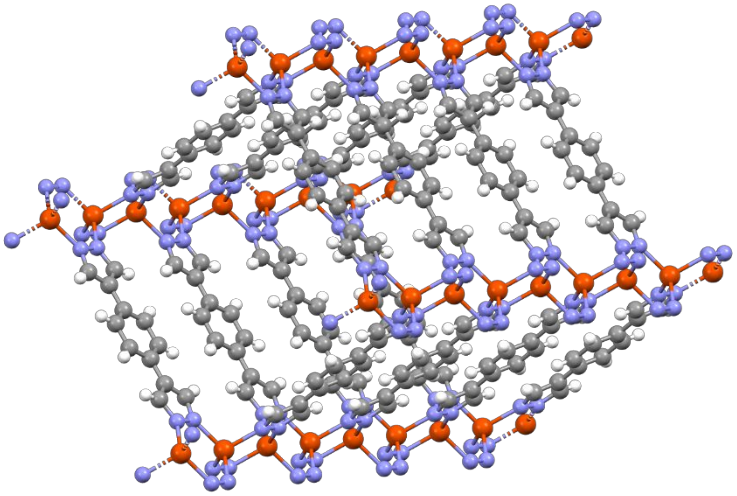

Figure S1. 3D snapshot of the Fe(bdp) framework. Orange, blue, gray, and white spheres represent Fe, N, C, and H atoms, respectively.

Structures

All the studied structures and a sample input file are available at the following github page: https://github.com/hnbabaei/Flexible-MOFs.git.

Green-Kubo calculation of thermal conductivity

The Green-Kubo relation for thermal conductivity is,

$$k_{ij} = \frac{V}{k_B T^2}\int_0^\infty \langle J_i(0)J_j(t)\rangle dt,\ i\ and\ j = x, y, z. \quad (1)$$

In which the *ij*-th element of the thermal conductivity tensor (k_{ij}, *i* and *j* = *x*, *y*, or *z*) at temperature *T* is calculated by integrating over time the heat current autocorrelation function (HCACF). The HCACF is extracted from equilibrium molecular dynamics (MD) simulations. In Eq. (1), k_B is the Boltzmann constant, *V* is the volume of the simulation box that contains the system of particles and ***J***(t) is the microscopic heat current.

The microscopic heat current is calculated from

$$\boldsymbol{J}(\mathrm{t}) = \frac{1}{V}\left[\sum_{i=1}^{N} E_i \mathbf{v}_i - \frac{1}{2}\sum_{i=1}^{N}\sum_{j \neq i}^{N} (\mathbf{F}_{ij} \cdot \mathbf{v}_j)\mathbf{r}_{ij}\right] \quad (2)$$

where $\mathbf{v}_i$ and E_i are the velocity vector and instantaneous energy of particle *j*. The quantities $\mathbf{r}_{ij}$ and $\mathbf{F}_{ij}$ are the displacement vector and interacting force between particles *i* and *j*. The parameter *N* is the total number of particles.

The heat current autocorrelation functions were calculated using a correlation time of 50 ps and then averaged and integrated for the thermal conductivity prediction. For all cases, we performed this procedure for four simulations starting from random velocity distributions. Thermal conductivity values were obtained from the plateau region of the HCACF integral that corresponded to 10 ps (the 10–20 ps time window). The error, which was calculated using thermal conductivity values from MD simulations at various initial conditions, was within 10%. To ensure that the supercell size used in the simulations (4×4×4) was sufficient, convergence tests were also performed on 3×3×3 and 6×6×6 supercells, and all values were within 5% of the average thermal conductivity determined using the 4×4×4 supercell.

This approach has been used previously for other MOFs, with results that are consistent with experimental measurements. For example, predicted thermal conductivities for the framework HKUST-1 under vacuum and loaded with various guests were consistent with experimental results using frequency-domain thermoreflectance (FDTR) and time-domain thermoreflectance (TDTR) measurement techniques on single crystals of HKUST-1 (see H. Babaei, M. E. DeCoster, M. Jeong, Z. M. Hassan, T. Islamoglu, H. Baumgart, A. J. H. McGaughey, E. Redel, O. K. Farha, P. E. Hopkins, J. A. Malen and C. E. Wilmer, *Nat. Commun.*, 2020, **11**, 4010).

Additionally, this approach has been used for MOF-5, with results consistent with experimental hot-wire measurements (see B. L. Huang, A. J. H. McGaughey, and M. Kaviany, *Int. J. Heat Mass Transf.*, 2007, **50**, 393–404 and B. L. Huang, Z. Ni, A. Millward, A. J. H. McGaughey, C. Uher, M. Kaviany and O. Yaghi, *Int. J. Heat Mass Transf.*, 2007, **50**, 405–411).

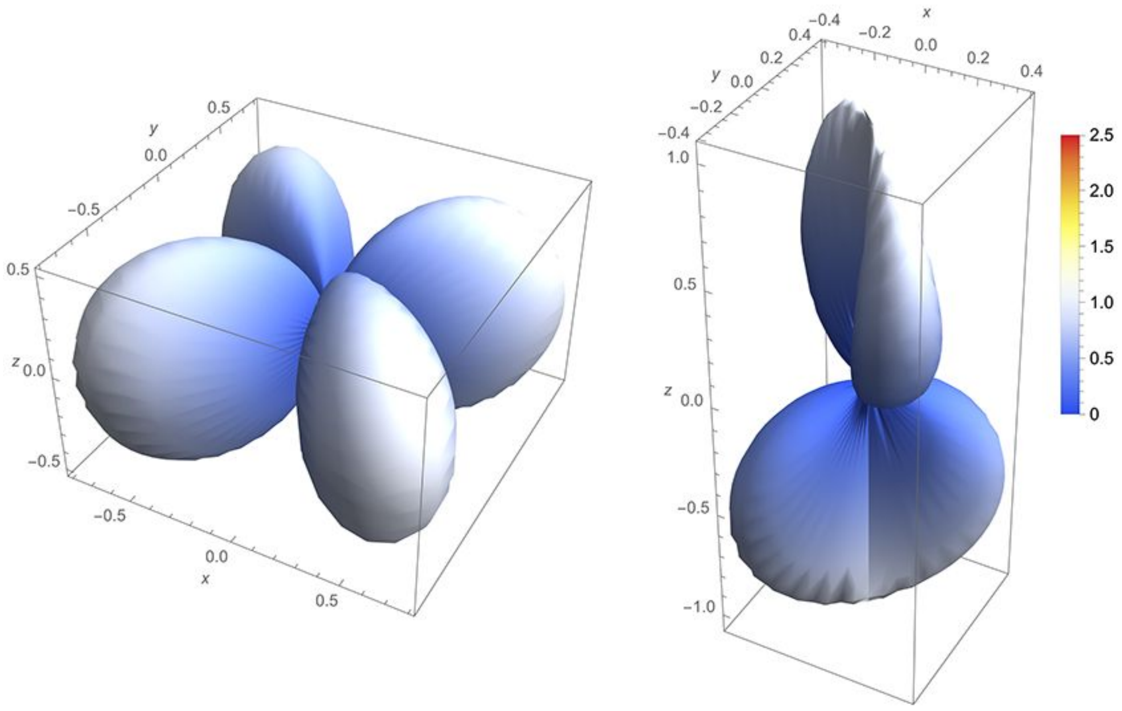

Figure S2. Calculated three-dimensional contours of directionally dependent off-diagonal elements k_{xy} (left) and k_{yz} (right) of the thermal conductivity tensor for contracted Fe(bdp), transformed by the transformation matrix, as described in the text. The radial distances and colors represent thermal conductivity magnitudes. Thermal conductivities are normalized by the diagonal element of thermal conductivity tensor of expanded Fe(bdp) in the x direction, k_{xx} = 4.6 W/mK.

Table S1. Unnormalized and normalized values of thermal conductivity for expanded and contracted Fe(bdp). All the off-diagonal elements are zero in the original *xyz* directions.

	**Unnormalized thermal conductivity (W/mK)**		**Normalized thermal conductivity**	
	Expanded	Contracted	Expanded	Contracted
k_{xx}	4.6	10.5	1	2.3
k_{yy}	4.6	0.63	1	0.13
k_{zz}	0.78	0.78	0.17	0.17
